# Lung image segmentation via generative adversarial networks

**DOI:** 10.3389/fphys.2024.1408832

**Published:** 2024-08-16

**Authors:** Jiaxin Cai, Hongfeng Zhu, Siyu Liu, Yang Qi, Rongshang Chen

**Affiliations:** ^1^ School of Mathematics and Statistics, Xiamen University of Technology, Xiamen, China; ^2^ School of Computer and Information Engineering, Xiamen University of Technology, Xiamen, China

**Keywords:** image segmentation, lung image analysis, machine learning, deep learning, generative adversarial networks, image processing

## Abstract

**Introduction:**

Lung image segmentation plays an important role in computer-aid pulmonary disease diagnosis and treatment.

**Methods:**

This paper explores the lung CT image segmentation method by generative adversarial networks. We employ a variety of generative adversarial networks and used their capability of image translation to perform image segmentation. The generative adversarial network is employed to translate the original lung image into the segmented image.

**Results:**

The generative adversarial networks-based segmentation method is tested on real lung image data set. Experimental results show that the proposed method outperforms the state-of-the-art method.

**Discussion:**

The generative adversarial networks-based method is effective for lung image segmentation.

## 1 Introduction

Machine learning (Nian et al., 2020; Shehab et al., 2022) has received substantial research interest due to its wide application in fields such as computer vision (Servadei et al., 2020), communication (Zhu et al., 2020), speech recognition (Latif et al., 2023), automatic medical diagnosis (Latif et al., 2020; Richens et al., 2020), and natural language processing (Chowdhary and Chowdhary, 2020). Deep learning (Cai et al., 2024) has recently become one of the most popular research topics in machine learning. Increasingly people have focused on deep learning-based image analysis, including image super-resolution (Wang et al., 2021a), image generation (Jiang et al., 2021), and image content classification (Onishi et al., 2020). Generative adversarial network (GAN) (Goodfellow et al., 2014) is a popular deep learning model in the research field of computer vision. It has become one of the most valuable technologies for image generation.

Medical image segmentation plays an important role in computer-aided clinical diagnosis and treatment (Habuza et al., 2021). Traditional medical image segmentation algorithms mainly include binarization methods (Seo et al., 2020), watershed algorithms (Chen et al., 2020), level set-based methods (Wang et al., 2021), semi-supervised clustering (Chen et al., 2023; Xu et al., 2023), and optimization models (Bateson et al., 2021). However, these algorithms achieve good results only when there is a large difference between the background area and the object area. The effect of image processing is poor when the background is similar, so other methods are needed to improve the segmentation.

To improve the accuracy of segmentation, curvature constraints and local feature constraints are often added to the models (Han et al., 2020; Wang et al., 2021c). However, these methods remain ineffective when distinguishing object areas that closely resemble the background. Therefore, the use of deep learning algorithms for medical image segmentation has become a research direction with theoretical significance and practical application value (Aref et al., 2021). Deep learning uses multilayer networks and learns rich local features. It has a strong fitting ability but also has some shortcomings such as a time-consuming training process and difficulty of explaining concretely. Deep learning-based medical image segmentation usually adopts a fully convolution neural network (FCN) (Chen et al., 2022), Mask R-CNN (Tu et al., 2023), U-Net (Deb and Jha, 2023), and other deep networks. The advantage of these models is that they have multilayer structures to generate rich features that help improve recognition performance. The disadvantages of these models are that the training time is often very long, and the segmentation results do not contain enough spatial details. Sometimes it cannot be guaranteed that the segmentation effects of them show a great improvement over traditional algorithms (Jia et al., 2023).

The lung is a frequent site of human diseases, and lung imaging constitutes a primary diagnostic tool for physicians. However, conventional images often contain excessive and irrelevant information. During diagnosis, these irrelevant areas can lead to inefficiencies and prolonged evaluation times. Lung image segmentation aims to expedite the extraction of relevant lung areas, thereby enhancing diagnostic efficiency for medical professionals (Gugulothu and Balaji, 2024). The purpose of lung image segmentation is to help doctors quickly extract the region of interest to extract biomarkers and help image-guided surgery. Gordaliza et al. (2018) proposed an unsupervised CT lung image segmentation model based on fuzzy connectedness for *mycobacterium* tuberculosis-infected patients. Xue et al. (2010) proposed a joint registration and segmentation algorithm to segment lung CT images for lung cancer diagnosis. Chen et al. (2019) proposed a lung CT image segmentation method by integrating an eigenvector space shape prior model and sparse shape composition. The approach can accurately discover pathological lung surfaces on low-dose CT images. Grychtol et al. (2010) proposed a lung electrical impedance tomography image segmentation method based on fuzzy logic. For the segmentation of lung images, Chen and Zhuang (2002) proposed an automatic segmentation method for lung parenchyma in chest high-resolution CT images. This method is divided into six steps: binarization, extracting upper and lower torso boundaries and removing the outer part of the torso, median filtering, connected domain marking, morphological filtering, mask display, and most of them are completed by machines. Zhao et al. (2006) proposed an interactive manual lung nodule segmentation method based on multi-slice CT images. This method can obtain ideal segmentation results through improved Live Wire algorithm for 3D reconstruction, but the segmentation speed depends on manual operation. Arthi et al. (2023) proposed a combination of denseNet and genetic algorithm for lung CT scans cancer classification. Although this algorithm takes a lot of time for segmentation, it can make the segmentation result more accurate. However, lung images have the characteristics of complex structures, low image quality, multimodality, gray level fuzziness, and uncertainty, which lead to an unsatisfactory segmentation effect for many algorithms (Chen et al., 2019). Especially, pulmonary nodules are very small. Some nodules are even hidden. Once the segmentation details are not well processed, these nodules will be hard to detect by traditional deep learning models (Cai and Zhu, 2019a).

GAN is a network developed by Goodfellow et al. (2014). GAN employs an algorithm that achieves the optimization goal through repeated antagonistic games (Goodfellow et al., 2014). GAN has been used for pulmonary nodule classification. Xu et al. (2018) introduced the derivative model of GAN deep convolution generation countermeasure network (DCGAN) into early pulmonary nodule classification. Messay et al. (2015) rated the benign and malignant grades of pulmonary nodules as five grades: benign, suspected benign, unknown, suspected malignant, and malignant. Xu et al. (2018) used improved DCGAN to classify pulmonary nodules based on this standard. However, how to perform lung image segmentation by GAN is still a challenge. GAN is also mainly used to expand images. It can change a certain number of pictures into many pictures of the same type. GAN can also achieve the transformation between different styles of pictures. Many researchers have used these algorithms to perform many interesting operations such as art painting. This inspired us to solve the lung image segmentation problem by employing GAN. In this paper, the original image and the segmented image of the lung are regarded as pictures of different styles for conversion.

This paper proposes a segmentation method based on GAN. We employ the Image-to-Image Translation with Conditional Adversarial Networks (Pix2Pix) (Isola et al., 2017) network, a variety of GAN, to adopt lung images. Pix2Pix is an image translation algorithm that can transform the blurred image into the exact image. This inspired us to perform image translation for image segmentation. Pix2Pix has demonstrated outstanding performance in various image-to-image translation tasks, including medical image segmentation, image denoising, and image restoration. Pix2Pix utilizes a conditional GAN framework, which allows it to learn a direct mapping from input images to target images using paired training data. This capability is particularly advantageous for segmentation tasks, where accurate mapping between input and output images is crucial. Pix2Pix compared to other methods such as U-Net. U-Net is specifically designed for biomedical image segmentation and has an encoder-decoder structure, while Pix2Pix can better capture the complex relationships in image-to-image translation tasks by exploiting adversarial loss. Pix2Pix is more suitable for paired image transformation tasks like ours. We employ Pix2Pix to translate the lung image to the segmented image. As an extension of our previous conference presentation (Cai and Zhu, 2019a), our methodology considers the original lung image (Kevin, 2017) as a blurred image and the segmented image as the exact image. The exact image refers to the binary segmentation result, where each pixel in the image is classified as either foreground (representing the region of interest) or background. This binary segmentation provides a clear delineation of the structures within the CT image that are relevant for further analysis. We translate the blurred image into the exact image using the Pix2Pix framework. Pix2Pix is a generative adversarial network (GAN) designed for image-to-image translation tasks. By training the network on pairs of blurred and exact images, the model learns to produce accurate binary segmentation results from the input CT images. The result of the translation from blur image to exact image is taken as the segmentation result. The Pix2Pix based segmentation method is tested on the lung image data set. Experimental results demonstrate that this method is effective and has better performance than the state-of-the-art method of medical image segmentation, the U-Net (Deb and Jha, 2023) architecture. This method offers the advantage of continuously improving accuracy with increasing training samples and iterations, while also achieving efficient segmentation post-training. Compared with the results of manual segmentation by experts, our algorithm is better at processing lung CT images. The contribution of this paper includes:

(1) We have introduced a novel method for lung image segmentation based on Pix2Pix, innovatively framing the lung segmentation task as a translation process from blurred to precise images. In this perspective, the original lung grayscale image is regarded as the “blurred” input, while the segmented image is considered the “exact” output. Our contribution lies in optimizing the application of GANs in lung CT image segmentation, particularly in handling complex image features and improving segmentation accuracy.
(2) The experimental results show that our method can achieve good results and outperforms the state-of-the-art method. Through fine-tuning the network architecture and optimizing the training strategy, we have not only improved segmentation accuracy but also made breakthroughs in handling challenging tasks such as processing blurred boundaries. The training time and test time of our method are also less than that of the state-of-the-art method.

The rest of this paper is organized as follows. Section 2 is the method. Section 3 is the experimental results and analysis. Section 4 is the discussions. Section 5 is the conclusion.

## 2 Materials and methods

### 2.1 Materials

**FIGURE 1:**
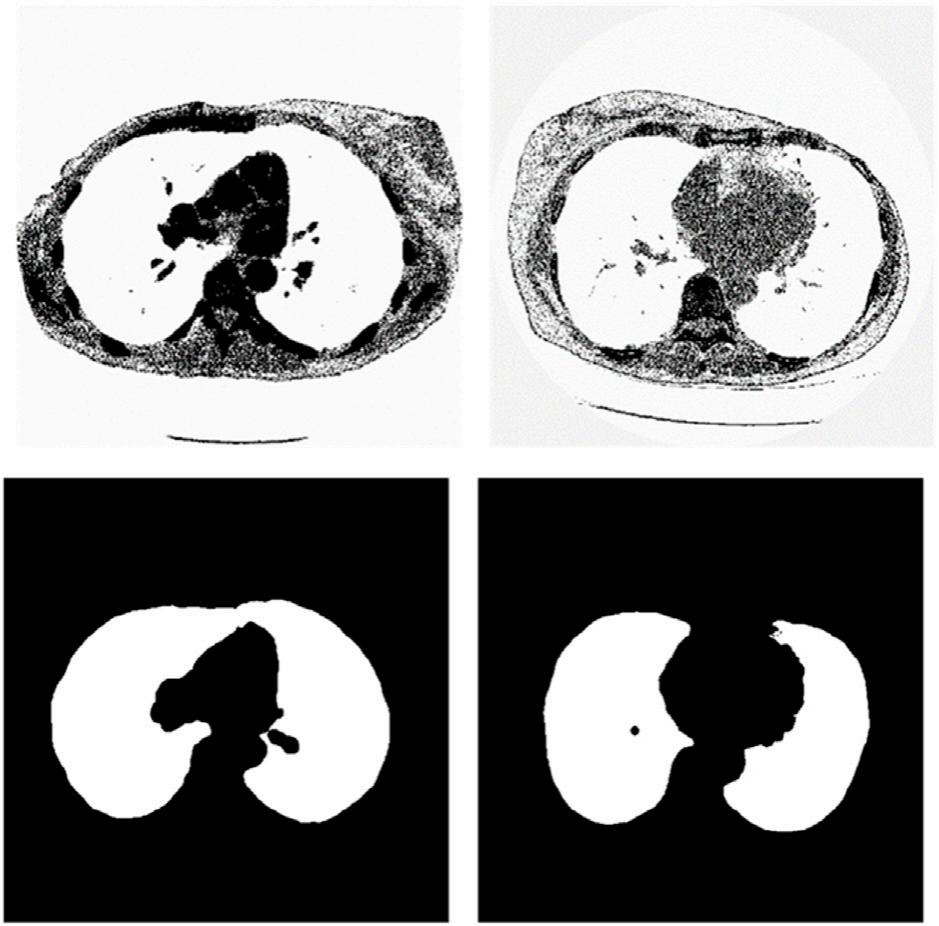
Examples of training lung CT images and their corresponding ground truths.

**FIGURE 2:**
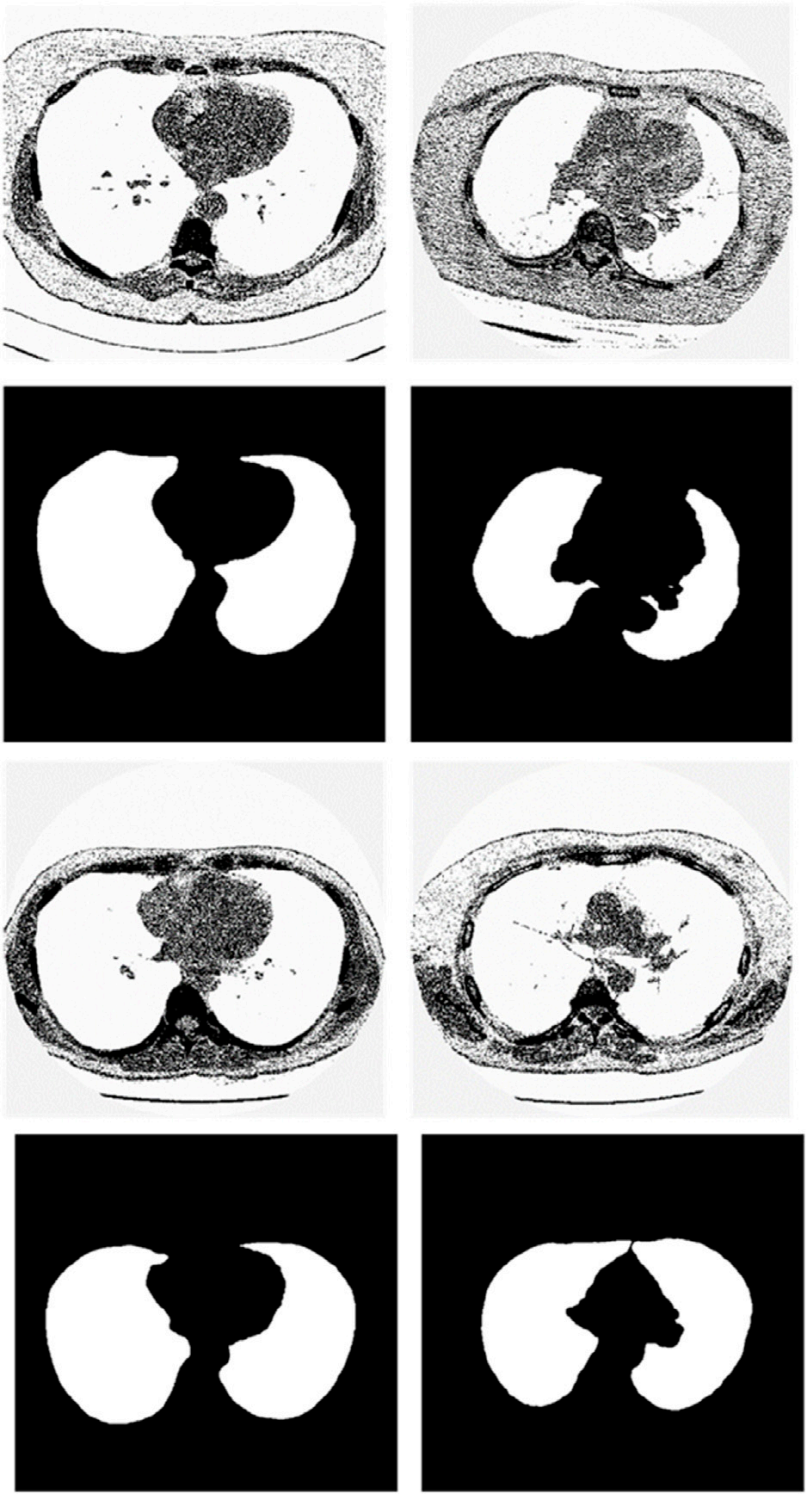
Examples of test lung CT images and their corresponding ground truths.

We chose the dataset “Finding_lungs_in_CT_data” (Kevin, 2017) for testing the methods. The dataset was published by Kevin Mader. The last update time is 2017, and the download time is March 2019. It had a set of manually segmented lung images. There are 2D and 3D images in the dataset. In this study, we did not use 3D images and only tested 2D images. There are 267 2D original gray-scale images, and 267 lung segmentation images corresponding to them one by one. The images belong to different patients. The dataset was divided by (Kevin, 2017) into a training set that has 237 lung images and a test set that contains 30 lung images. The manual segmentation results of experts were given by (Kevin, 2017). This lung CT image dataset uses lung window optimization, and the parameters are set to about 1600 HU for WW and −600 HU for WL, which effectively improves the contrast of lung details and is conducive to accurate observation. For the Pix2Pix model, 256 × 256 resolution training is used to improve the efficiency of the algorithm. Because artifacts and lung abnormalities may impair the recognition accuracy of deep learning models, this dataset has been strictly screened to eliminate low-quality images to ensure the purity of samples. The average pixel coverage of lung area is 60,846, corresponding to the actual area of about 43,590.55 square millimeters, accounting for 25.43% of the volume ratio of a single section, and the average HU value is −657.913, reflecting the typical density of lung tissue. Through the selection of data, the risk of misdiagnosis is effectively avoided, the accuracy and robustness of the lung anomaly detection model based on deep learning are enhanced, the recognition challenges caused by artifacts and anomalies are overcome, and the possibility of misdiagnosis is reduced. There are some manual segmentation errors in the ground true images due to the expert’s mistake, and these incorrect training labels may pose difficulties for artificial intelligence diagnosis, as incorrect training labels can train the artificial intelligence diagnosis to tend towards incorrect recognition. The details of the dataset are given in (Kevin, 2017). Examples of training images and their corresponding ground truth images are shown in Figure 1. Examples of test images and their corresponding ground truth images are shown in Figure 2.

### 2.2 Methods

GAN employs an algorithm to achieve the optimization goal through repeated antagonistic games. It consists of a generator and a discriminator. The main purpose of the generator is to generate enough false images so that the discriminator cannot determine whether these images are true or not. The purpose of the discriminator is to ensure that it is not deceived by the generator. The classical generative models usually need to give a specific framework and require parameter estimation which needs complex calculations such as Monte Carlo sampling or other approximate estimation procedures. Different from classical generative models, GAN does not need complex calculations about probability. Besides, GAN does not need to specify the distribution type. It directly stimulates the distribution of real data by deep neural networks. The gradient descent algorithm, typically using the Backpropagation (BP) algorithm, is usually employed in the training process.

The optimization function of GAN is written as Equation 1.

$$\min_{G}\max_{D}V\left(D,G\right)=E_{x∼P_{data\left(x\right)}}\log D\left(x\right)+E_{z∼P_{z}\left(z\right)}\log\left(1-D\left(G\left(z\right)\right)\right),$$ (1)

Where G is the generator, and D is the discriminator. x is the real picture. z is the noise input of the generator G . $G\left(z\right)$ is the picture generated by generator G . $D\left(x\right)$ is the probability that the discriminator D judges whether the real picture is true or not. $D\left(G\left(z\right)\right)$ is the probability that the discriminator judges whether the picture generated by the generator G is true or not.

The purpose of GAN is to learn the distribution of training data (Goodfellow et al., 2014). To accomplish this goal, first, noise is input into the generator. The generator transforms this noise into a picture. The discriminator identifies the simulated pictures with the real picture and gives the true and false coefficients of the image. Through cyclic alternate training, the generator and the discriminator are both improved. The generator can generate synthetic images that are very similar to the original images (Jia et al., 2023).

Pix2Pix (Isola et al., 2017) is a framework developed based on conditional GAN (cGAN) (Mirza and Osindero, 2014). Similarly, Pix2Pix has a generator G and a discriminator D . The input and output of G are both a single image. To ensure that the generated image matches the input image, the loss function of conditional GAN takes the form as Equation 2.

$$\Gamma \_cGAN\;\left(G,D\right)=E\_\left(x,y\right)\;[\log\;D\;\left(x,y\right)]+E\_\left(x,z\right)\;[\log\;\left(\;1-D\left(x,G\left(x,z\right)\right)\right),$$ (2)

Where G is the generator and D is the discriminator. z is the input random vector. x is the image to be converted. y is the target image. In the process of image translation, a lot of information is shared between the input and the output of the generator G . An L1 loss is added to ensure the similarity between the input image and the output image is large. The *L*1 loss function is written as Equation 3.

$$\Gamma_{L1}\left(G\right)=E_{x,y,z}\left[{\left\|y-G\left(x,z\right)\right\|}_{1}\right],$$ (3)

Finally, the loss function of Pix2Pix is constructed based on merging the cGAN loss function and the L1 loss function. The solution of Pix2Pix is written as Equation 4.

$$G^{*}=\arg\min_{G}\max_{D}\Gamma_{cGAN}\left(G,D\right)+\lambda \Gamma_{L1}\left(G\right),$$ (4)

**FIGURE 3:**
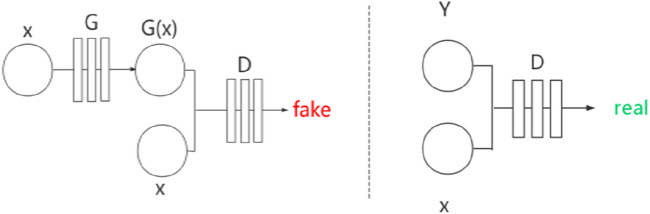
Network structure of Pix2Pix. X represents the input image, Y represents the ground truth image, $G\left(X\right)$ denotes the image generated by the generator network $G^{*}$ , and D represents the discriminator network.

Where $\Gamma_{c}$ denotes the cGAN loss function and $\Gamma_{L1}$ denotes the added L1 loss function. $G^{*}$ denotes the final solution. The structure of Pix2Pix is shown in Figure 3. In Figure 3, X represents the image to be converted, Y represents the real image, $G\left(X\right)$ represents the image generated by the generator, $G^{*}$ represents the generation network, and D represents the discrimination network.

**FIGURE 4:**
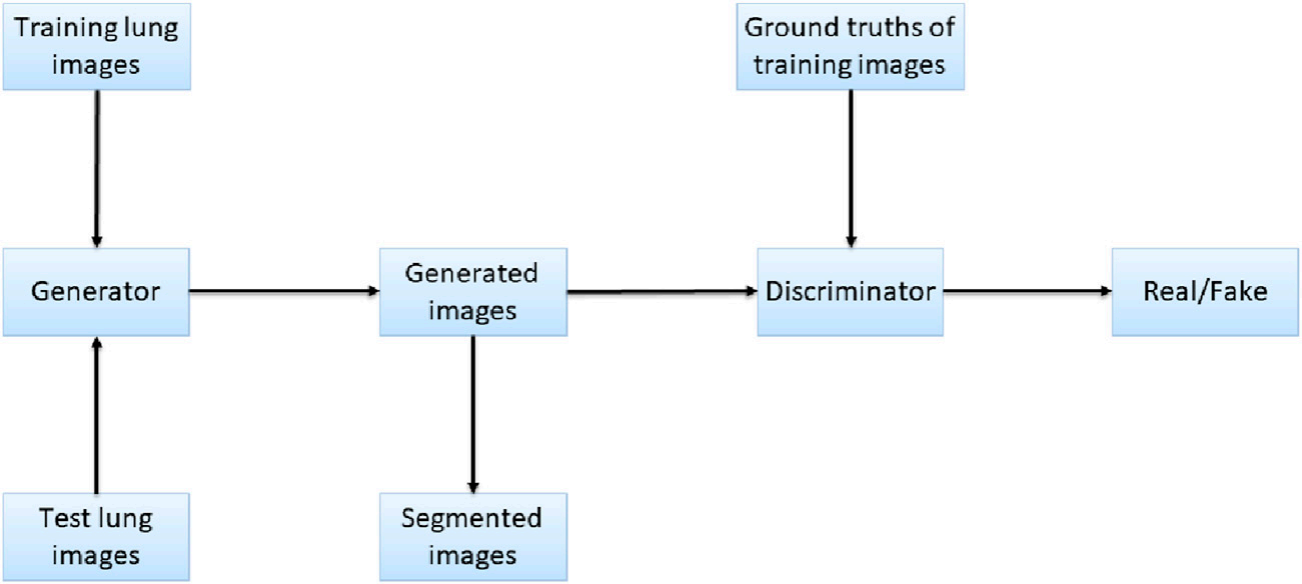
Workflow of the proposed method.

Pix2Pix is a model that can transform blurred images into exact images. We employed Pix2Pix to translate the original image to the segmented image. We took the original gray image of the lung as a blurred image and made the segmented image as an exact image. Then we translated the blurred image into the exact image by Pix2Pix. The result of the translation was taken as the segmentation result. Figure 4 shows the workflow of the method. In the training stage, the training images are input into the generator, and the generated images and the ground truths are input into the discriminator for judging. In the test stage, the test images are input into the generator, and the generated images are employed as the output segmentation results.

## 3 Results

### 3.1 Experimental environments

The experimental environments were Windows 10 OS, CPU i5-4210U @ 1.70 GHz, 8 GB memory, Anaconda 3 (64-bit), Spyder 3.3.3, Numpy package, Pillow package, Pytorch 0.4.0 package, and Torchvision 0.2.1 package. No GPUs were used for experiments.

### 3.2 Performance evaluation

In medical image processing, especially for the segmentation task of lung images, researchers often use the “accuracy” metric to evaluate the consistency between the model predictions and the true annotations. In the image segmentation scenario, a pixel can be viewed as an individual classification decision point, so indeed it is possible to think of accuracy as the classification correct rate at each pixel level. For the lung image segmentation task, a true positive means the model correctly segments a pixel to a foreground (lung) area outcome when the actual segmentation outcome is the foreground area. A false positive means the model segments a pixel to a foreground (lung) area outcome when the actual segmentation outcome is the background area. A true negative means the model correctly segments a pixel to a background area outcome when the actual segmentation outcome is the background area. A false negative means the model segments a pixel to a background area outcome when the actual segmentation outcome is the foreground (lung) area.

We used the overlap rate as a metric of lung area difference between real area and segmented area. The overlap rate reflects the degree of overlap between the segmented regions produced by the algorithm and the true labeled regions. It is usually computed by dividing the number of pixels in which the segmentation result intersects the true labeled region by the number of pixels in the union of the two. The ideal value of the overlap rate is 1, which means that the segmentation results are completely consistent with the true annotations.

F-measure is the harmonic mean of Precision and Recall, which combines information from both precision and recall and can provide a balanced evaluation. Precision refers to the fraction of pixels correctly segmented by the algorithm over all pixels marked as segmented by the algorithm, while recall refers to the fraction of pixels correctly segmented by the algorithm over all pixels that should be segmented.

The segmentation results of the test images were compared with the ground truth image which was manually segmented by experts. The accuracy, overlap rate, and F-measure were calculated. The computation formulas are listed as Equations 5–7.

$$Accuracy=\frac{TP+TN}{TP+TN+FP+FN}$$ (5)

$$Overlap\;rate=\frac{\left|A\wedge B\right|}{\left|A\vee B\right|}$$ (6)

$$F=2P\times \frac{R}{P+R}$$ (7)

Where TP is the true positive rate, and FP is the false positive rate. TN is the true negative rate, and FN is the false-negative rate. A denotes the segmentation result of Pix2Pix, and B denotes the image manually segmented by experts. P denotes the precision, and R denotes the recall. F denotes the F-measure.

### 3.3 Setting the training epoch number as 20

We used the whole training set which has 237 images to train the network. The training epoch number was set as 20. After the network was trained, the tested images were imported into the networks for generating the corresponding segmented images. The segmented images were compared to the manually segmented images. The accuracy, overrate and F-measure of all 30 test images were computed. Table 1 shows the segmentation performances of all 30 test images. From Table 1, we can see that the segmentation results of Pix2Pix approximate the ground truth. Table 1 shows the mean and standard deviation of the segmentation performance of the test images. As can be seen from Table 1, the average accuracy of Pix2Pix based lung segmentation is 89.64%. The range of accuracy is [54.79%, 96.77%], and the standard deviation of accuracy is 7.37%. The average overlap of Pix2Pix based lung segmentation is 93.03%. The range of overlap is [31.91%, 97.85%], and the standard deviation of overlap is 11.83%. The average F measure of Pix2Pix based lung segmentation is 95.85%. The range of F-measure is [48.38%, 98.91%], and the standard deviation of F-measure is 9.07%. Experimental results demonstrate that our proposed method is effective and achieves considerable performance.

**TABLE 1:** Statistical values of training and testing image segmentation performance when the number of training epochs is set to 20.

	Metric	Minimum	Maximum	Mean	Standard deviation
Test	Accuracy	0.54785	0.96773	0.8963389	0.07371077
	Overlap rate	0.31905	0.97852	0.9302968	0.11829190
	F-measure	0.48375	0.98914	0.9585260	0.09074364
Train	Accuracy	0.63797	0.96985	0.9013378	0.04818742
	Overlap rate	0.34268	0.98439	0.9514375	0.06152281
	F-measure	0.51044	0.99214	0.9737749	0.04290255

Table 1 also shows the mean and standard deviation of the segmentation performance of the training images. As can be seen from Table 1, the average training accuracy of Pix2Pix based lung segmentation is 90.13%. The range of training accuracy is [63.80%, 96.99%], and the standard deviation of training accuracy is 4.82%. The average training overlap of Pix2Pix based lung segmentation is 95.14%. The range of training overlap is [34.27%, 98.44%], and the standard deviation of training overlap is 6.15%. The average training F-measure of Pix2Pix based lung segmentation is 97.38%. The range of training F-measure is [51.04%, 99.21%], and the standard deviation of training F-measure is 4.29%. Experimental results show that there was little difference between the segmentation performances of the training images and those of the test images. Experimental results demonstrate that our proposed method has good performance.

### 3.4 Setting the training epoch number as 100

**FIGURE 5:**
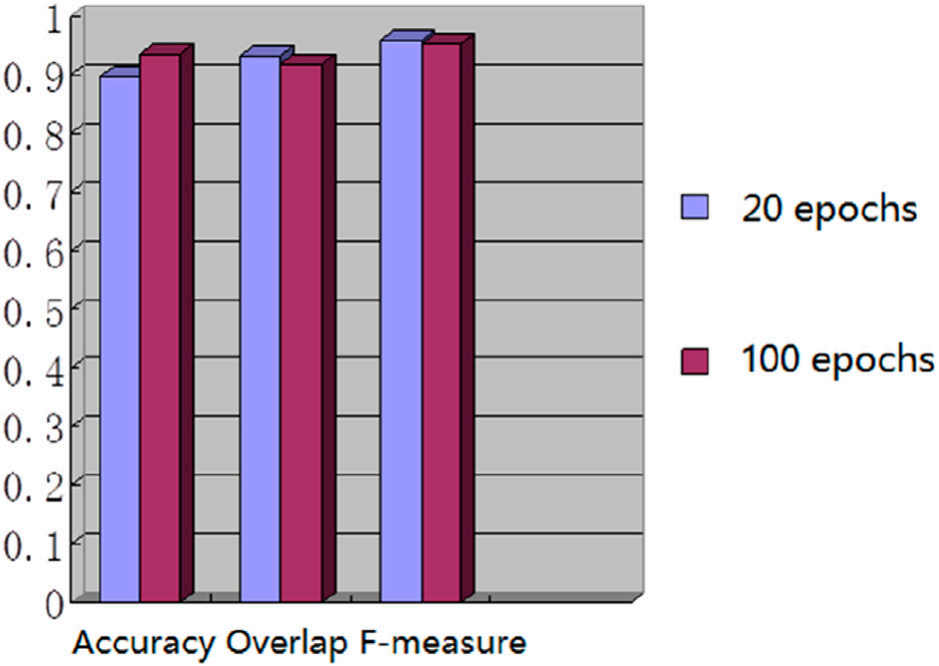
Comparison results of training with different epochs.

We used the whole training set which has 237 images to train the network. The training epoch number was set as 100. After the network was trained, the tested images were imported into the network for generating the corresponding segmented images. The segmented images were compared to the manually segmented images. The accuracy, overrate and F-measure of all 30 test images were computed. Table 2 shows the segmentation performances of all 30 test images. Table 2 shows the mean and standard deviation of the segmentation performance of the test images. As can be seen from Table 2, the average accuracy of Pix2Pix based lung segmentation is 93.40%. The range of accuracy is [56.13%, 96.01%], and the standard deviation of accuracy is 7.09%. The average overlap of Pix2Pix based lung segmentation is 91.69%. The range of overlap is [32.68%, 97.54%], and the standard deviation of overlap is 13.04%. The average F-measure of Pix2Pix based lung segmentation is 95.03%. The range of F-measure is [49.29%, 98.75%], and the standard deviation of F-measure is 9.70%. Compared to the experiment in which the training epoch number was set as 20, the average accuracy increases from 89.64% to 93.40%, the average overlap rate decreases from 93.03% to 91.69%, and the average F measure decreases from 95.85% to 95.03%. Figure 5 shows the comparison results between training 20 epochs and training 100 epochs. As can be seen from Figure 5, increasing the training epoch number from 20 to 100 has not significantly improved the segmentation performance.

**TABLE 2:** Statistical values of the segmentation performance of the tested image when the training epoch number was set as 100.

	Minimum	Maximum	Mean	Standard deviation
Accuracy	0.56131	0.96005	0.9340500	0.07088869
Overlap rate	0.32677	0.97539	0.9169011	0.13043104
F-measure	0.49258	0.98754	0.9503117	0.09669589

### 3.5 Setting the training sample number as 67 and the training epoch number was set as 20

**FIGURE 6:**
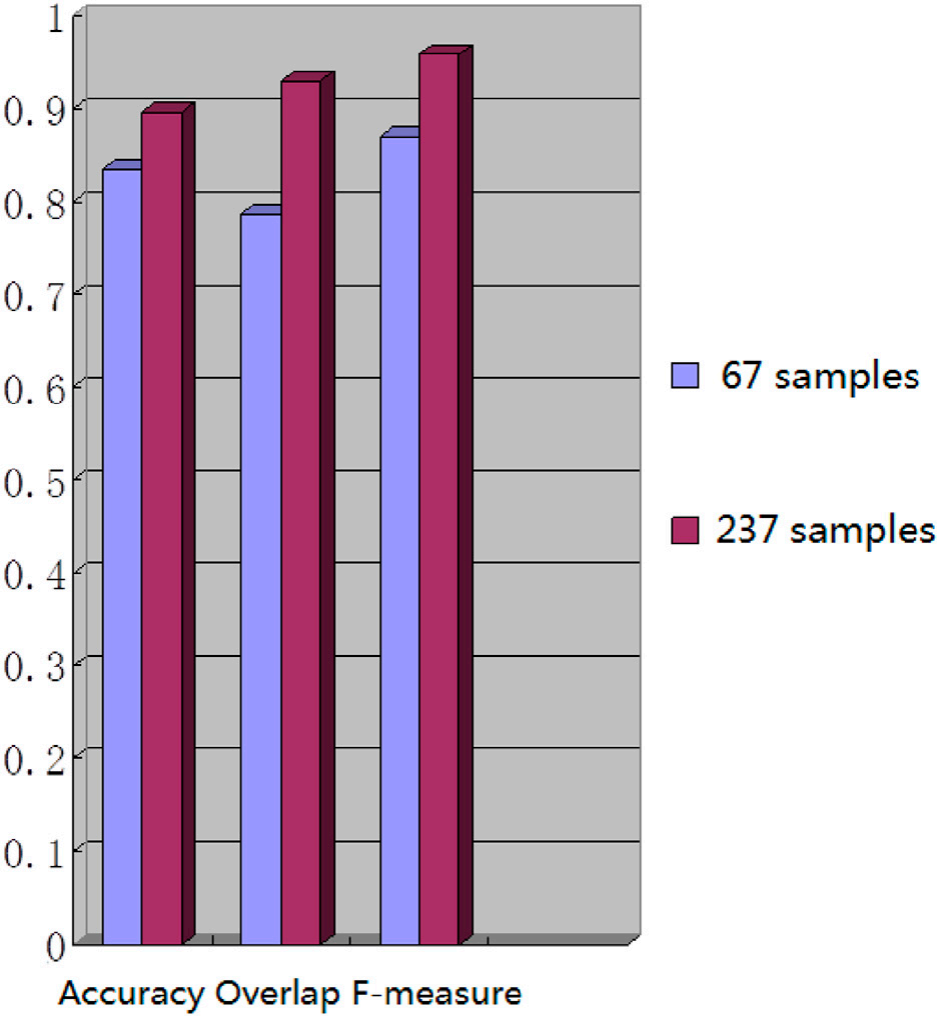
Comparison results of training with different samples.

We chose 67 training samples from the training set to train the networks. The training epoch number was set as 20. After the networks were trained, all the tested images were imported into the networks and generated the corresponding segmented images. The segmented images were compared to the manually segmented images. The accuracy, overrate and F-measure of all 30 test images were computed. Table 3 shows the segmentation performances of all 30 test images. Table 3 shows the mean and standard deviation of the segmentation performance of the test images. As can be seen from Table 3, the average accuracy of Pix2Pix based lung segmentation is 83.46%. The range of accuracy is [50.26%, 92.73%], and the standard deviation of accuracy is 8.09%. The average overlap of Pix2Pix based lung segmentation is 78.61%. The range of overlap is [34.42%, 94.80%], and the standard deviation of overlap is 15.66%. The average F-measure of Pix2Pix based lung segmentation is 87.05%. The range of F-measure is [51.22%, 97.32%], and the standard deviation of F-measure is 11.51%. Compared to the experiment in which the training samples were set as 237, the average accuracy decreased from 89.64% to 83.46, the average overlap rate decreased from 93.03% to 78.61%, and the average F-measure decreased from 95.85% to 87.06%. Figure 6 shows the comparison results between training with 237 samples and training with 67 samples when the training epoch number was set as 20. As can be seen from Figure 6, decreasing the training sample number from 237 to 67 has significantly hastened the decline of segmentation performance. However, using a smaller training set led to less training time. Table 4 shows the comparison of computation time. For the experiment in which the training sample number is 237 and the training epoch number is 20, the training time is 24 h. For the experiment in which the training sample number is 237 and the training epoch number is 100, the training time is 120 h. For the experiment in which the training sample number is 67 and the training epoch number is 20, the training time is 6 h. For the experiment in which the training sample number is 237 and the training epoch number is 20, the computation time for testing the whole test set is 2 min and 25 s, and the computation time for testing the whole training set is 19 min and 23 s.

**TABLE 3:** Statistical values of the segmentation performance of the tested lung images when the training sample number was 67 and the training epoch number was set as 20.

	Minimum	Maximum	Mean	Standard deviation
Accuracy	0.50266	0.92731	0.8345856	0.08098433
Overlap rate	0.34424	0.94779	0.7860806	0.15662531
F-measure	0.51217	0.97319	0.8705305	0.11506310

**TABLE 4:** Comparison of computation time.

	Training 20 epochs with 237 samples (h)	Training 100 epochs with 237 samples (h)	Training 20 epochs with 67 samples (h)	Testing 30 samples (s)	Testing 237 samples (s)
Computation time	24	120	6	2min25	19min23

### 3.6 Test samples with large F-measure

**FIGURE 7:**
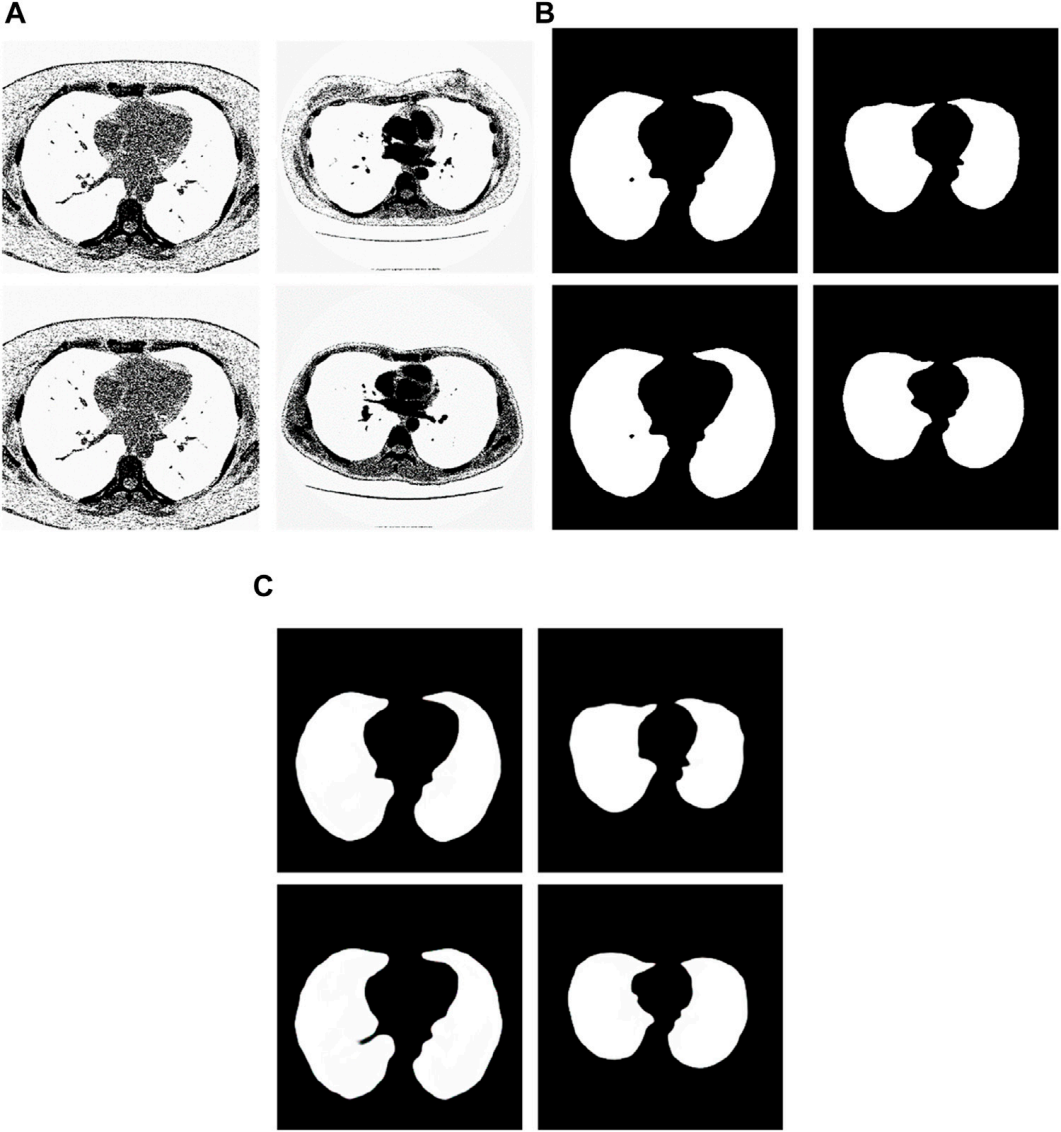
Examples of the test images with high F-measure and their experimental results. **(A)** shows the test lung images. **(B)** shows the corresponding ground-truth images. **(C)** shows the corresponding segmentation results by the proposed method.

We have analyzed the test samples which had the largest F-measure in the whole test set (Abdlaty et al., 2021). The results are shown in Figure 7. Figure 7A shows the tested lung images. Figure 7B shows the corresponding ground truth images. Figure 7C shows the segmentation results. The first figure of Figure 7A is the test image whose ID is ID_0246_Z_0228. Its corresponding segmentation result is shown in the first figure of Figure 7C. The segmentation was performed by using the Pix2Pix trained 20 epochs with 237 training samples. The F-measure is 0.98914, which is the largest F-measure among that of the test images segmented by the Pix2Pix which was trained 20 epochs with 237 training samples. The second figure of Figure 7A is the test image whose ID is ID_0247_Z_0070. Its corresponding segmentation result is shown in the second figure of Figure 7C. The segmentation was performed by using the Pix2Pix trained 100 epochs with 237 training samples. The F-measure is 0.98754, which is the largest F-measure among that of all test images segmented by the Pix2Pix which was trained 100 epochs with 237 training images. The third figure of Figure 7A is the test image whose ID is ID_0246_Z_0228. Its corresponding segmentation result is shown in the third figure of Figure 7C. The segmentation was performed by using the Pix2Pix trained 20 epochs with 67 training images. The F measure is 0.97319, which is the largest F measure among that of all test images segmented by the Pix2Pix which was trained 20 epochs with 67 training images. The fourth figure of Figure 7A is the test image whose ID is ID_0154_Z_0070. Its corresponding segmentation result is shown in the fourth figure of Figure 7C. The segmentation was performed by using the Pix2Pix trained 20 epochs with 237 training images. The F-measure is 0.99214, which is the largest F-measure among that of all training images segmented by the Pix2Pix which was trained 20 epochs with 237 training images. From Figure 7, we can see that our segmentation results are like the results manual segmented by experts.

### 3.7 Test samples with small F-measure

**FIGURE 8:**
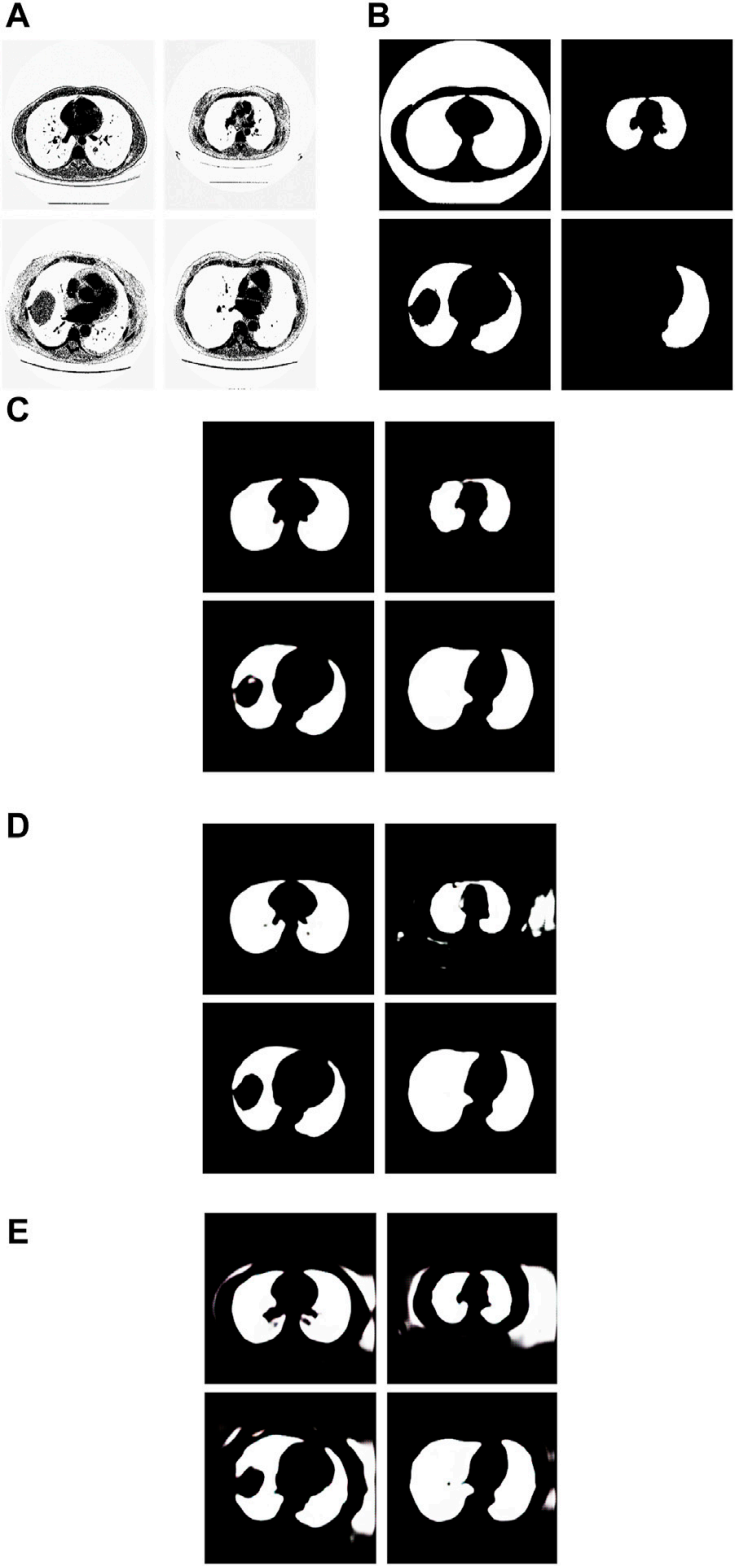
Examples of the test images with small F-measure and their experimental results. **(A)** shows the test lung images, **(B)** shows the corresponding ground-truth images, **(C)** shows the segmentation results of the Pix2Pix model trained on 237 samples with 20 epochs, **(D)** shows the segmentation results of the Pix2Pix model trained on 237 samples with 100 epochs, and **(E)** shows the segmentation results of the Pix2Pix model trained on 67 samples with 20 epochs.

We have analyzed the test samples which had the smallest F-measure in the whole test set. The results are shown in Figure 8. Figure 8A shows the tested lung images. Figure 8B shows the corresponding ground truth images. Figure 8C shows the segmentation results by the Pix2Pix trained 20 epochs with 237 samples. Figure 8D shows the segmentation results by the Pix2Pix trained 100 epochs with 237 samples. Figure 8E shows the segmentation results by the Pix2Pix which was trained 20 loops with 67 samples.

The first figure of Figure 8A is the test image whose ID is ID_0254_Z_0075. Its corresponding segmentation result is shown in the first figure of Figure 8C. The segmentation was performed by using the Pix2Pix which was trained 20 epochs with 237 training images. The F-measure is 0.48375, which is the smallest F-measure among that of all test images segmented by the Pix2Pix which was trained 20 loops with 237 training images. However, it can be seen that the segmentation is effective. From the first figure of Figure 8B we can see that the ground truth image got from experts is very poor. The reason that the F-measure is very small is that the ground truth image used to compare has a big mistake. We can see that the Pix2Pix segmentation has outperformed the manual segmentation by experts.

The second figure of Figure 8A is the test image whose ID is ID_0241_Z_0124. Its corresponding segmentation result is shown in the second figure of Figure 8D. The segmentation was performed by using the Pix2Pix trained 100 loops with 237 training images. The F-measure is 0.74140, which is the smallest F-measure among that of all test images segmented by the Pix2Pix which was trained 100 loops with 237 training images. The reason that the F-measure is very small is that training 100 loops caused the overfitting. However, as shown in the second figure of Figure 8C, the segmentation result by the Pix2Pix which was trained 20 loops with 237 training images is very good.

The third figure of Figure 8A is the test image whose ID is ID_0243_Z_0056. Its corresponding segmentation result is shown in the third figure of Figure 8E. The segmentation was performed by using the Pix2Pix trained 20 loops with 67 training images. The F-measure is 0.68454, which is the smallest F-measure among that of all test images segmented by the Pix2Pix which was trained 20 epochs with 67 training images. The reason that its F-measure is very small is that there were not enough training samples. In Figure 8, the expert mistakenly manually segmented the lung images due to a mistake. The expert only segmented the right half of the lung, without processing the left half of the lung. In addition, experts did not completely remove the background during manual segmentation. As a result, the model cannot fully learn the complexity of the lung structure, particularly in distinguishing the boundary between the background and lung tissue. So, the background removal is not thorough in the segmentation results of our method. However, our method successfully segmented the other half of the lung that the expert overlooked for segmentation. In contrast, as depicted in the second figure of Figure 8C and the second figure of Figure 8D, the segmentation results produced by Pix2Pix, which was trained using 237 training images, demonstrate high quality. However, as shown in the second figure of Figure 8C and the second figure of Figure 8D, the segmentation results by the Pix2Pix which was trained with 237 training images are very good.

The fourth figure of Figure 8A is the image whose ID is ID_0079_Z_0072. This is a special image that belongs to the training set and the test set simultaneously. Its corresponding segmentation result is shown in the fourth figure of Figure 8C. The segmentation was performed by using the Pix2Pix trained 20 loops with 237 training images. The F-measure is 0.51044, which is the smallest F-measure among that of all training images segmented by the Pix2Pix which was trained 20 loops with 237 training images. However, the segmentation results are still good. From the fourth figure of Figure 8B we can see that the ground truth image got from experts is very poor. The reason that the F measure is very small is that the ground truth image used to compare has a big mistake. And the Pix2Pix segmentation has outperformed the manual segmentation by experts.

### 3.8 The segmentation results by the proposed method when the wrong manual segmentation results were given by experts

**FIGURE 9:**
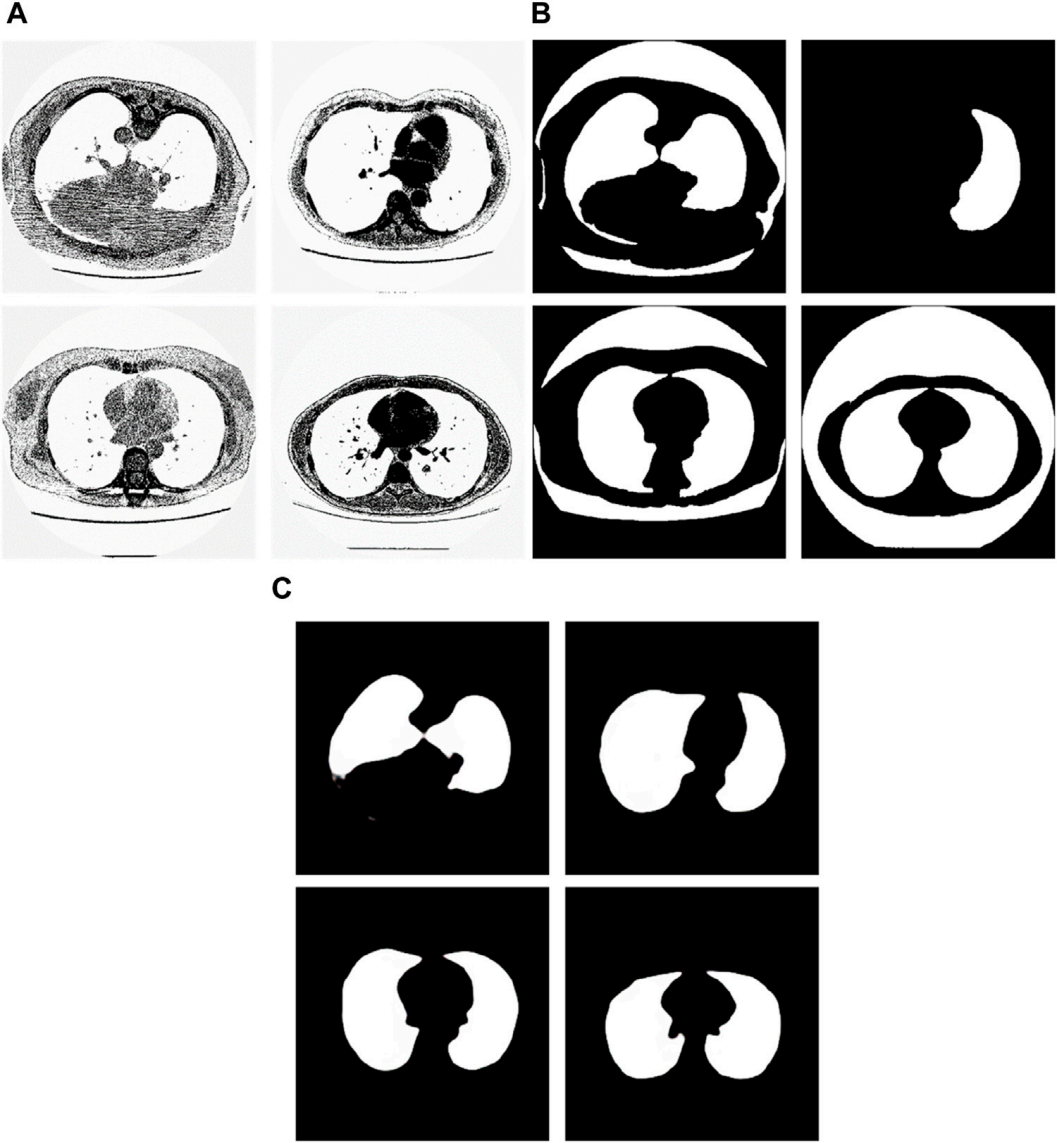
Examples of the test images whose segmentation results by the proposed method are better than the corresponding manual segmentation results by experts. **(A)** shows the tested images, **(B)** shows the corresponding ground-truth images labeled by experts, and **(C)** shows the corresponding segmentation results obtained by the Pix2Pix model trained on 237 samples with 20 epochs.

From the examples in Figure 8, we can see that the proposed method is sometimes able to correct the manual segmentation results by experts. When the experts made the wrong segmentation, the proposed method can still get the right segmentation. Figure 9 shows more such examples. Figure 8A shows the tested lung images. The first figure of Figure 8A is the test image whose ID is ID_0052_Z_0108. The second figure of Figure 9A is the test image whose ID is ID_0079_Z_0072. The third figure of Figure 8A is the test image whose ID is ID_0134_Z_0137. The last figure of Figure 9A is the test image whose ID is ID_0254_0075. Figure 9B shows their corresponding ground truth images. From Figure 9B we can see that the experts made the wrong segmentation results. Figure 9C shows the corresponding segmentation results by the Pix2Pix trained 20 epochs with 237 samples. From Figure 9C we can see that the Pix2Pix segmentation can correct the wrong manual segmentation by experts. In Figure 9, the expert also mistakenly manually segmented the lung images. The expert also only segmented the right half of the lung, without processing the left half of the lung. In addition, experts also did not completely remove the background during manual segmentation. However, our method successfully removed redundant background and successfully segmented the other half of the lung that was missed by expertise. Our method has successfully overcome the impact of manual labeling errors.

### 3.9 Comparison with state-of-the-art methods

It has been proven that deep learning-based segmentation outperforms traditional segmentation methods such as region growing for lung segmentation. Traditional image segmentation methods such as edge detection and thresholding are commonly used in image processing tasks. Edge detection algorithms, like the Canny edge detector (Triwibowo et al., 2023), identify the boundaries of objects in an image by detecting areas with significant intensity changes. These methods are computationally simple and effective for images with clear boundaries, but they are sensitive to noise and may struggle with complex images. Thresholding methods, such as Otsu’s method (Vite-Chávez et al., 2023), segment images by selecting a threshold value that separates the foreground from the background. While effective for images with distinct contrast, thresholding methods may not perform well on images with complex intensity distributions. Machine learning based segmentation methods, such as U-Net, have shown significant improvements in segmentation performance. U-Net is a convolutional neural network that consists of an encoder-decoder structure with skip connections that retain high-resolution features. It is particularly effective for medical image segmentation tasks and can achieve high accuracy with a relatively small amount of labeled data. However, U-Net requires substantial computational resources. Besides, U-net has been proven as the state-of-the-art method for deep learning-based lung segmentation and outperformed FCN and Mask R-CNN (Chen et al., 2018; Kohl et al., 2018; Tong et al., 2018; Park et al., 2020). We choose U-Net, a state-of-the-art architecture in the field of deep learning, as well as classical non-machine-learning segmentation methods including the Otsu thresholding technique and Canny edge detection algorithm, as the compared methods. We compare the pix2Pi-based segmentation with the segmentation based on U-Net, Otsu thresholding technique, and Canny edge detection algorithm on the lung dataset. In this study, the U-Net we used follows the standard architectural configuration, including convolutional layers, pooling layers, up-sampling layers, and skip connections. To ensure the fairness of the comparison, we did not make any additional architectural modifications or hyperparameter tuning to U-Net but used the widely accepted default settings. Furthermore, we trained and tested all algorithms under the same hardware and software environment to ensure the consistency of experimental conditions. 67 samples in the training set were used for training, and the whole test set was tested. The training epoch number was 20. The average accuracy with standard deviation, the average overlap rate with standard deviation, and the average F-measure with a standard deviation of the two algorithms were compared. We have also compared the training time and the test time of the two machine learning methods. The comparison results are shown in Table 5. The comparison results show that the performances of Otsu threshold technology and the Canny edge detection algorithm are far worse than those of the Pix2Pix model. Pix2Pix has a better segmentation effect. Compared to U-Net, Pix2Pix costs less computation time and has better segmentation accuracy, overlap rate, and F-measure. The Pix2Pix takes half of the training and test time of U-Net, which indicates that the Pix2Pix algorithm is more time-efficient than U-Net.

**TABLE 5:** Comparison results of machine learning and non-machine learning models.

	Accuracy	Overlap rate	F-measure	Training time	Test time
Pix2Pix	0.835 ± 0.081	0.786 ± 0.157	0.871 ± 0.115	6 h	2 min25 s
U-Net	0.820 ± 0.070	0.690 ± 0.109	0.811 ± 0.085	12 h	6 min12 s
Otsu	0.413	0.199	0.311	-	-
Canny	0.735	0.110	0.198	-	-

## 4 Discussions

One of the drawbacks of this paper is that we only test lung segmentation. In the future, we intend to apply this method to segmentation tasks involving other organs, such as the pancreas with its indistinct boundaries, or blood vessels characterized by their slender and intricate nature.

Our work was first released on the preprint website Arxiv (Cai and Zhu, 2019b) and conference (Cai and Zhu, 2019a) in 2019. There are several relative works about lung segmentation using GAN. In 2020, Munawar et al. (2020) proposed the use of GAN to perform lung segmentation on chest X-ray images. They use the generator of GAN to generate a segmented mask of chest X-ray image and use the discriminator of GAN to distinguish the ground truth and the generated mask. Our work is different from this work. Our work uses a conditional GAN with L1 loss to produce a more discriminative segmentation on CT images. In 2021, Pawar and Talbar (Pawar and Talbar, 2021) proposed the LungSeg-Net for automatic lung segmentation of lung CT scans. The input lung CT images are encoded to a set of feature maps by the trail of encoders. Our work is different from it. We directly transform the input lung CT images to a segmented image without a supplementary feature map generation step. In 2021, Tan et al. (2021) proposed the LGAN, a Lung CT scan segmentation method by generative adversarial network. The LGAN used EM distance for pixel segmentation, while our work uses a simple L1 distance which is easy to calculate to reduce computational complexity. In 2021, Tyagi and Talbar (2022) proposed the CSE-GAN, a 3D conditional generative adversarial network for lung nodule segmentation. The LGAN used EM distance for pixel segmentation, while our work uses a simple L1 distance which is easy to calculate to reduce computational complexity. Our work is different from this work. Our work employs a 2D conditional generative adversarial network with sparse loss term for 2D lung field segmentation without squeeze and excitation procedure with huge computational burden. There are also some current works on using GAN for other lung image analysis tasks such as lung CT image synthesis, lung cancer CT image generation, classification of lung cancer disease, and 3D lung tumor reconstruction. In 2021, Jiang et al. (2021) proposed a conditional generative adversarial network for lung CT image synthesis. In 2020, Jiang et al. (2021) proposed a combination of convolutional neural network and generative adversarial network for lung CT pulmonary nodule classification. Compared to (Onishi et al., 2020; Jiang et al., 2021), our wok focus on application of conditional generative adversarial network on lung region segmentation on CT images.

In this study, we experimented with different numbers of training images (237 or 67) and epochs (20 or 100) to optimize the Pix2Pix segmentation network. These specific choices were made due to limitations in dataset size and computational resources. We acknowledge that this is not an exhaustive investigation and that our conclusions are preliminary. Future work will involve a more extensive exploration of training parameters to fully optimize network performance. Nonetheless, the current results provide valuable insights and a foundation for further research.

The strengths of our research methodology are evident in its ability to continuously enhance accuracy with increasing training samples and iterations while maintaining a relatively short segmentation process duration. Experimental data validates these capabilities: after 20 training iterations with 67 samples, the average F-measure reached 0.871 ± 0.115; reducing the training samples to 20 under the same iteration count significantly elevated the average F-measure to 0.959 ± 0.091; and with 100 samples, the average F-measure stabilized at 0.950 ± 0.097. These series of experiments robustly demonstrate that augmenting the number of training samples markedly improves the precision of lung image segmentation. In comparison to manual segmentation by experts, our algorithm exhibits superior performance in handling specific images. Nonetheless, despite the model’s exceptional performance, segmentation errors may still occur when dealing with complex images, often attributed to inherent image complexity and limitations in the training dataset. To further enhance the model’s generalization capability and segmentation accuracy, future research endeavors will focus on refining training strategies and optimizing dataset construction methods. These efforts aim to achieve comprehensive and precise segmentation outcomes in both clinical applications and broader contexts.

The experimental results show that Pix2Pix based lung segmentation outperforms the manual segmentation by experts many times. However, the experts can easily correct those mistakes when they notice they have made the wrong segmentation.

## 5 Conclusion

This paper proposed a lung segmentation method using Pix2Pix. The Pix2Pix was employed to translate the original lung image into the segmented image. The Pix2Pix segmentation method was tested on the real lung image data set. Experimental results show that the proposed method is effective and outperforms the state-of-the-art methods.

## Data Availability

The original contributions presented in the study are included in the article/supplementary material, further inquiries can be directed to the corresponding author.
